# Multidisciplinary Care for Moebius Syndrome and Related Disorders: Building a Management Protocol

**DOI:** 10.3390/jcm13113309

**Published:** 2024-06-04

**Authors:** Amar Odedra, Wendy Blumenow, Jennifer Dainty, Soumit Dasgupta, Susana Dominguez-Gonzalez, Jose Gonzalez-Martin, Helen Hartley, Maria Kelly, Victoria H. McKay, Ravi Sharma, Stefan Spinty, Adel Y. Fattah

**Affiliations:** 1 Regional Paediatric Burns and Plastic Surgery Service, Alder Hey Children’s NHS Foundation Trust, Liverpool L12 2AP, UK; 2 Department of Speech and Language Therapy, Alder Hey Children’s NHS Foundation Trust, Liverpool L12 2AP, UK; wendy.blumenow@alderhey.nhs.uk; 3 Department of Psychology, Alder Hey Children’s NHS Foundation Trust, Liverpool L12 2AP, UK; 4 Department of Audiology, Alder Hey Children’s NHS Foundation Trust, Liverpool L12 2AP, UK; 5 Department of Orthodontics and Paediatric Dentistry, Alder Hey Children’s NHS Foundation Trust, Liverpool L12 2AP, UK; 6 Department of Ophthalmology, Alder Hey Children’s NHS Foundation Trust, Liverpool L12 2AP, UK; 7 Therapy Department, Alder Hey Children’s NHS Foundation Trust, Liverpool L12 2AP, UK; 8 Department of Genetics, Liverpool Women’s Hospital, Liverpool L8 7SS, UK; 9 Ear Nose and Throat Department, Alder Hey Children’s NHS Foundation Trust, Liverpool L12 2AP, UK; 10 Department of Paediatric Neurology, Alder Hey Children’s NHS Foundation Trust, Liverpool L12 2AP, UK

**Keywords:** Moebius syndrome, congenital hereditary facial palsy, cranial dysinnervation disorders, multidisciplinary management

## Abstract

Moebius syndrome is a collection of orofacial anomalies with highly variable features affecting many different systems but characterised by bilateral facial palsy and absent eye abduction. We largely regard Moebius syndrome as a diagnosis of exclusion. Lack of awareness and knowledge means that children often fall between services, leading to treatment delays and difficulty interfacing with social care and schools, with long-term impact on physical health and psychosocial development. We developed a multidisciplinary team comprising core clinicians (lead physician, geneticist, speech and language therapist, psychologist and specialist nurse) and an expanded group to encompass the other affected systems. The interactions between our specialties lead to the development of a treatment protocol, which we present. The protocol harnesses the aspects of care of children with a range of other rare diseases at a specialised paediatric centre and synthesises them into a holistic approach for MBS and related conditions. Management is sequenced on an “ABC-style” basis, with airway, feeding, vision and speech taking priority in the early years. We define management priorities as airway stabilisation with swallow assessment, ocular surface protection and maintenance of nutritional support. Management principles for issues such as speech, reflux, drooling and sleep issues are outlined. In later years, psychological support has a prominent role geared towards monitoring and interventions for low mood, self-esteem and bullying.

## 1. Introduction

Moebius syndrome (MBS) is a disorder of the brainstem with a heterogeneous aetiology [1,2]. It has a variable presentation, generally regarded as specifically affecting abducens (VI) and facial (VII) nerve function as well as other cranial and extracranial features. There is considerable overlap with other conditions including myopathic and cranial dysinnervation disorders [3]; this has led to difficulties in defining the syndrome. There are no widely agreed diagnostic criteria, but we recently published a systematic review [4] that documented clinical features (Table 1) and determined two main groups labelled with the diagnosis. We have previously published the need for a multidisciplinary approach [5]. This article presents our protocol for management of this population (Scheme 1).

## 2. Scheme 1—Management Protocol

### 2.1. At Presentation

#### 2.1.1. Management Priorities

Stabilise airway and ensure safe swallow.

Ensure ocular surface protection.

Maintain nutritional support and weight gain.

#### 2.1.2. Diagnostic and Screening Investigations

MRI to determine brain stem morphology and look for associated brain anomalies that may point to future issues.

Ophthalmic assessment.

Echo: cardiac defects are known to occur in 33% of patients.

WGS (if available) and phenotyping assessment.

Myopathy screen.

Videofluoroscopy if choking when feeding or repeated chest infections.

Photography of all clinical signs.

#### 2.1.3. Institute Supportive Measures

Airway strategies and supplementary feeding, ocular lubrication, etc.

## 3. Ongoing Management

### 3.1. From 6 Months

Medial rectus botox as indicated.

Institute dental assessment and brushing at first erupted tooth.

### 3.2. From 18 Months

Informal speech assessment including feeding video.

Consider strabismus surgery.

Developmental screen (ASQ).

### 3.3. From 3 Years

Formal speech assessment including feeding video.

Developmental screen (ASQ and parent questionnaire/interview).

Clinical photography.

### 3.4. 5 Years

Clinical photography.

Psychology assessment (DSQ: if concerns are highlighted, consider administering the Wechsler Pre-school and Primary Scale of Intelligence [WPPSI-IV]).

### 3.5. From 6 Years

Smile reconstruction surgery as indicated.

Begin facial therapy in those that would benefit.

### 3.6. 8 Years

Formal vestibular assessments and rehabilitation.

Audiometry.

CT labyrinth if concerns.

### 3.7. 9 Years (Pre-High School)

SDQ (strengths and difficulties questionnaire and if indicated, Wechsler Intelligence Scale for Children [WISC-V]).

Photography.

### 3.8. 13 Years (Mid-High School)

SDQ (strengths and difficulties questionnaire and if indicated, Wechsler Intelligence Scale for Children [WISC-V]).

### 3.9. 16 Years (Prior to High School Graduation)

SDQ (strengths and difficulties questionnaire and if indicated, Wechsler Adult Intelligence Scale for Children [WAIS-V]).

### 3.10. 20 Years

Genetic counselling.

Transition to adult services if appropriate.

Photography.

## 4. Initial Assessment

**Figure 1:**
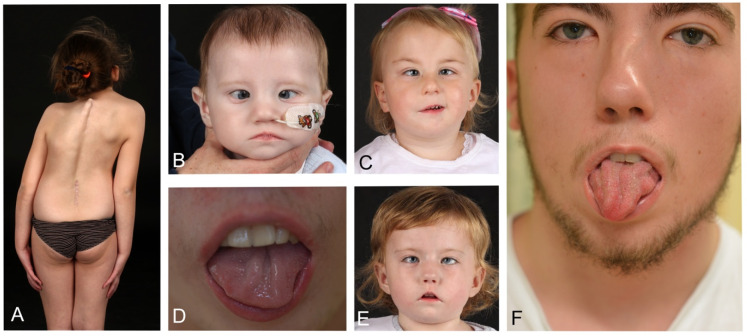
Spectrum of Moebius syndrome features. (**A**) Scoliosis. (**B**) Expressionless face, esotropia, epicanthic folds feeding difficulties (tube fed). (**C**) Bilateral VI palsy, unilateral VII palsy. (**D**) Rugose hypoplastic tongue. (**E**) Characteristic expressionless face, epicanthic folds, short upper lip bilateral VI palsy (esotropia) and bilateral VII (facial) palsy. (**F**) Rugose tongue, bilateral VI and VII palsy following strabismus surgery.

**Figure 2:**
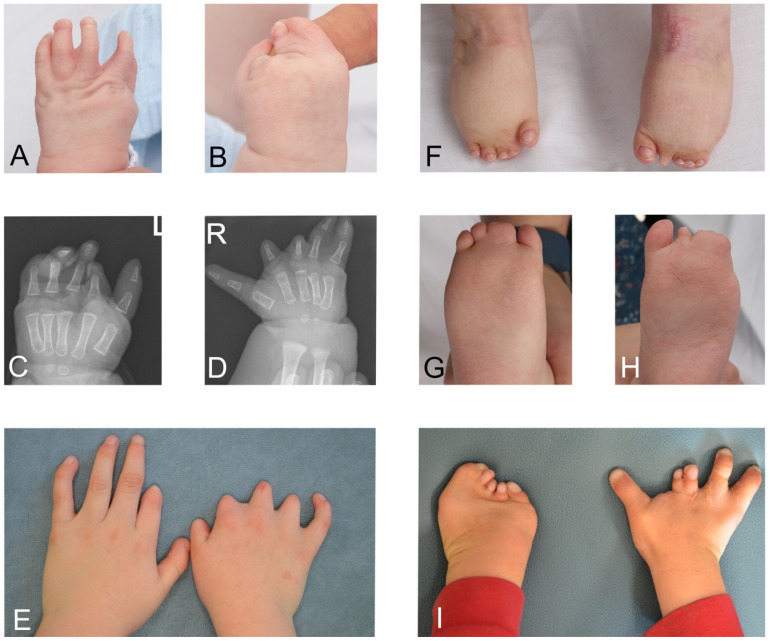
Limb anomalies in Moebius syndrome. (**A**–**D**) Photo and radiographs of right and left hands of patient with bilateral symbrachydactyly. (**E**) Bilateral symbrachydactyly. (**F**–**H**) Hypoplastic toes, talipes and syndactyly of feet. (**I**) Bilateral complicated symbrachydactyly.

Initial assessment is based on an “ABC” method, but depends on the age at which the patient is first diagnosed. Frequently, diagnosis is delayed and suggested by parents to physicians after researching the internet. Clinical findings are summarised in Table 1 and Figure 1 and Figure 2.

## 5. Airway

This is most relevant to neonates; lower cranial nerve palsies (IX to XII) occur in 40–50% and micrognathia in 75% of cases: both may compromise airway protection. 10/449 cases required long-term ventilation due to apnoeas and central breathing issues and 20/449 cases died of apnoeas, aspiration or aspiration-pneumonia [4]. This highlights that feeding/swallowing and airway management are closely linked and should be monitored carefully.

Those with airway obstruction should be transferred to a neonatal high dependency unit for oxygen monitoring and airway support. Emergency airway management in the newborn should first consider positioning of the neonate, the indications for nasopharyngeal airway include increased airway difficulties during feeding (most common), obstruction when handled or obstruction not alleviated by positioning. Emergency endotracheal intubation for acute obstruction is rare, and long-term airway issues require tracheostomy [6].

## 6. Feeding

Multiple cranial nerve palsies affect oral motor skills, including lip closure, tongue movement, chewing and swallowing function. In the neonate, the ability to latch is severely reduced, leading to significant feeding difficulties and poor weight gain. As the child develops, these palsies limit the ability of the child to move food in their mouth causing difficulty manipulating the food bolus to swallow safely. Oral incontinence is exacerbated by poor lip seal due to the facial palsy and manifests as difficulty in sucking, keeping food in the oral cavity and using a straw. Children are observed using their fingers to move food around their mouth and push it posteriorly to help trigger a swallow reflex. If abnormal, coughing, choking and aspiration can occur.

Initial assessment is directed to early feeding and swallow assessment with videofluoroscopy if indicated, to establish swallow safety using a variety of different consistencies. If aspiration occurs, texture modification can be used to slow the food flow and help the child co-ordinate oral movement. If the swallow is safe and the issue is suction and/or oral motor difficulties then strategies like those for cleft palate patients can be used: One can attempt cup-feeding, soft bottles and different teats, under the supervision of an experienced speech and language therapist (SALT).

If there is failure to thrive in spite of the above conservative measures, an orogastric tube should be placed to avoid obstruction of the small-diameter nasal passages in obligate nasal breathers. This can be replaced by a nasogastric tube if there are no airway concerns. After approximately 6 weeks, if there is no improvement in weight gain or oral feeding, then a gastrostomy is considered: this is our preferred option for long-term feeding difficulties [7] and is considered as a positive step towards oral feeding whilst not compromising weight gain. Specifically, the gastrostomy is considered to reduce the degree of oral aversion (an issue in this population) and facilitate re-establishing oral feeding [8,9].

Oral motor skills and swallow function are assessed at relevant time points individualised for each patient and in liaison with local SALT services. If function improves with growth, then tube feeding is modified to establish a hunger pattern and oral stimulation with food is used to prevent oral aversion. Eventually, the aim is to use standard strategies to wean the child from the tube feed. When weaning, modified utensils such as flat spoons are important, as the lips cannot seal over the curved surface of a normal spoon.

In older children, the oral feeding may need to be modified to account for poor oral motility. Texture modification of foods is important, as foods that require significant chewing or very flaky/crumbly foods are often difficult for these children to manage.

## 7. Vision

The key issues are corneal protection, strabismus and occasionally anaesthetic cornea (V_1_ nerve palsy); visual anomalies are unusual [1]. All children with MBS require an ophthalmologic assessment of corneal integrity, acuity and eye movements. In most cases, the Bell phenomenon (upward rolling of the eyes with attempted blink) is intact and corneal ulceration does not occur. In fact, the Bell phenomenon is the blink mechanism due to the limited eyelid closure as a consequence of the facial palsy. If there is red eye or irritation, then preservative-free ocular lubrication should be started immediately with prompt referral to an ophthalmologist. Orthoptic assessment is performed as soon as the child is cooperative; eye movement patterns may exclude alternate diagnoses (Table 2) [10].

Apart from corneal protection the other main issue is the esotropia due to the unopposed action of the medial rectus (Figure 1). Over time, this muscle contracts making subsequent surgery more difficult. To counteract this, botulinum toxin injections relax the medial rectus and facilitate future strabismus surgery [11]. Such surgery is for cosmetic purposes as established esotropia causes amblyopia. Corneal sensation should also be tested and if anaesthetic, referral to a centre that can provide corneal neurotisation [12] to re-establish sensation is recommended.

## 8. Auditory Assessment

Hearing is critical for development of speech and the audiovestibular system may be affected in MBS [13,14] with conductive deafness or sensorineural dysfunction in up to 30% [4]. Audiovestibular investigations can commence as early as 1 year of chronological age but best results are obtained when the child is older (approximately 6–8 years) [15]. All children in the UK have a neonatal (objective) hearing test (otoacoustic emissions) as part of universal newborn hearing screening programme and if abnormalities are detected additional testing (e.g., auditory brainstem response) is indicated: https://www.gov.uk/government/publications/newborn-hearing-screening-programme-nhsp-operational-guidance/6-patient-journey-from-screen-to-referral (accessed on 4 April 2024). Behavioural testing relies on the child to communicate their *perception* of hearing (a cognitive task) and multiple factors in MBS may confound this. For this reason, a low threshold for objective testing and monitoring of expressive and receptive language skills will allow for interventions before hearing loss can contribute to speech delay (see Supplementary Materials) [16]. Assessment of the vestibular system has not been studied in MBS, but deficits may contribute to poor motor coordination and difficult navigation skills [17].

## 9. Speech and Language Development

Speech development relates to the production of intelligible speech whereas language development refers to the cognitive processes around communication. Issues are three-fold: facial palsy hampers lip seal, whilst tongue and poor oromotor coordination hampers articulation, leading to compensatory sounds and finally, cognitive issues can exacerbate difficulties in communication. Facial palsy affects lip mobility and the presence of tongue atrophy (Figure 1) and fasiculations can significantly affect tongue movement and placement for differentiating between speech sounds. Both effect the articulation of a child and therefore speech development. Specifically, achieving lip closure for bilabial sounds, e.g., /*p*//*b*/ and /*m*/; and tongue placement for many sounds including /*t*//*d*//*n*/ and /*s*/. However, other issues that can also be present include delays in language development (receptive and expressive language), hearing difficulties and delays in cognitive development/learning ability. MBS children can also have difficulties with self-esteem and confidence, which can affect their communication skills. Consideration should also be given to other aspects of communication that may be limited in a child with MBS, for example, facial expression, pitch and tone of voice.

Assessment and intervention for these children should start young and intervention style/technique may change as the child develops. Baseline hearing assessments are recommended (above). Protocols should include assessment of oral motor skills (those not related to making speech sounds), speech (articulation and phonological progress) and receptive/expressive language skills. Resonance may also need to be assessed on an individual basis if required [18]. Time points for assessment should be individualised and are somewhat dictated by the age the child is referred (Scheme 1). It has proved useful to use video assessment and analysis with MBS [19], using this as a baseline and for comparison of oral motor and speech skills over time. There is no specific assessment system that is particularly good for MBS; we recommend using the tools that are routine in your current practice.

Most early assessments are managed informally, i.e., by observing the child in a play situation, whilst monitoring verbal and non-verbal interaction, play skills, social communication development and general interaction skills. At this early age, direct and formal assessment is not possible; however, standardised parental questionnaires can be conducted with parents/carers to give an idea of level of function with receptive and expressive language. More formal assessments can successfully be carried out after about the age of 3, although this varies from child to child. The types of standardised assessments used include those that look specifically at speech production and those that look at core language skills—both receptive and expressive. Furthermore, specific assessments can be carried out if particular areas of deficit are found. These are all on the premise that the child has the skill levels to sit for a formal assessment, which can take from 30 to 60 min depending on what is being assessed. In the situation where this is not the case, we would rely on informal observation as above, parental reporting, information from school and home videos.

Many children with MBS do develop intelligible speech; however, there is a subgroup that will rely on Augmentative and Alternative Communication (AAC), this may include pictures, gesture/signing and pointing or more complex computerised systems. It is therefore important that early SALT intervention includes introduction of early skills required for AAC, e.g., use of gesture, pictures, and cause and effect. Each child will be assessed on an individual basis as to the type of communication aid that suits them best. For some, having knowledge of sign language is sufficient, although this is not always appropriate for the MBS population if limb abnormalities are present, nonetheless, is often the first option explored. Signing is very functional in environments where those around the child are familiar with signing, e.g., in school and in the home environment. As children get older, if they are reliant on or use as an adjunct to speech, other forms of AAC can be considered using electronic devices/apps on phones. There are very bespoke assessment units where the most appropriate type of device can be advised with support around funding for them.

Intervention may include a variety of different approaches. Some children may benefit from direct therapy targeting oral motor skills and/or articulation therapy; this would require significant commitment and time from all involved, including SALT, school/nursery and parent/carers (see Supplementary Materials). Systems such as TalkTools^®^ (www.talktools.com) employ standard techniques and special tools to structure oral placement therapy—their efficacy in MBS remains to be determined. By contrast, cued articulation where the child uses signals to indicate certain sounds is only as good at the child’s language skills and the listener’s knowledge of the technique. Regardless of the approach to therapy, an integrated pathway in both the home and educational setting is essential, with excellent coordination between all teams involved.

## 10. Dental Management

MBS patients commonly have hypoplastic teeth and their primary dentition exfoliates slowly. They may exhibit missing teeth (primary and permanent). Additionally, they may be incapable of closing their mouth and have predisposition to viscous saliva, which together with the dry-mouth and dental hypoplasia makes them highly susceptible to caries. Therefore, it is essential to initiate a preventative dental program as soon as the first tooth erupts [20,21]. Normal childhood dental reviews and dental care are essential due to higher risk of dental problems.

During mixed dentition, an orthodontist should monitor occlusal development and facial growth. Orthodontic treatment should be provided according to the age and type of malocclusion. Different individual orthodontic treatments have been described in the literature, including orthognathic surgery at skeletal maturity to tackle the long face and micrognathia, and improve the occlusion and the facial proportions [22].

## 11. Reflux

Reflux is common in MBS and approached in the same way as non-MBS patients. First, efforts should be made to ensure feeding takes place upright and feeds adequately spaced throughout day as to not overfeed. Alginates (Gaviscon^®^) are first line in treating reflux and should be mixed with milk feeds. If symptoms persist, thickening agents such as Carobel^®^ can be used. Health practitioners should follow their normal protocols; proton pump inhibitors, e.g., omeprazole, are used in severe cases and pro-kinetics, e.g., domperidone, should be generally reserved for the most severe cases due to concerns regarding safety and efficacy in some groups [23].

## 12. Drooling

Drooling is a common finding [4] due to inadequate lip closure and the impaired swallow: Moebius children produce normal volumes of saliva that pools due to poor movement and subsequently overflows. But in the context of the facial difference of MBS, drooling can further exacerbate their psychosocial wellbeing and is therefore important to tackle [5,24].

SALT assessment is vital and management should be approached in a stepwise fashion (Table 3) [25].

Efforts to improve posture and a programme of exercises to stimulate sensation improve jaw closure, lip and tongue movements can be effective. Behavioural therapies conditioning patients to swallow or wipe saliva have been indicated to improve oral awareness and initiate swallow but may be difficult to encourage in those with impaired cognition [26].

Hyoscine is available as a convenient transdermal patch lasting up to 3 days. However, it has a significant side effect profile, particularly a dermal reaction to adhesive [27] and its long-term efficacy largely unreported. We use glycopyronium bromide as a first line; it is easier to titrate and well tolerated. Thereafter, a hyoscine patch can be considered. If medical management falters, botulinum toxin injections into the salivary glands are an effective treatment [28]. The effects on oral hygiene should be monitored.

## 13. Neurological and Neuromuscular Assessment

All children suspected to be affected by MBS, especially those with additional clinical signs beyond those listed (“Moebius-plus”) should be evaluated by a paediatric neurologist. The main differential diagnoses are rare congenital neuropathies or myopathies (Table 2). Congenital myotonic dystrophy, fascio–scapulo–humeral dystrophy (FSH) or myasthenia gravis can present with severe facial weakness in the neonatal period. Clinical evaluation includes a thorough antenatal history including exposure to trauma, toxins or infection in the early stages of the pregnancy. A full system examination including a detailed evaluation of cranial nerves, muscle bulk, tone and power, deep tendon reflexes and plantar response, any signs of dystonia, spasticity or other abnormal movements, joint contractures, torticollis or scoliosis, is required. Nerve conduction studies and electromyogram may be performed to help define the nerves involved. MRI brain (and in selected cases, CT to detect calcifications), might demonstrate structural abnormalities of the brainstem, hypoplasia or absence of affected cranial nerves. MRI in neonates, due to their small size is of less value in detecting minor abnormalities but is used to rule out significant brain structural abnormalities. In children with abnormal muscle tone, muscle power or joint contractures, scanning of the spinal cord might be indicated. Further investigation to determine the cause for the clinical presentation, for example a muscle biopsy, are guided by electrophysiology and neuro-imaging results. Recently, modified diffusion tensor MRI imaging has been suggested to discriminate MBS from other congenital facial palsy disorders [29].

A constellation of upper and lower limb anomalies occur in MBS (Figure 2) and such anomalies are managed by an MDT specialised in the care of such differences and includes plastic and orthopaedic surgeons, occupational and physiotherapists. Gross motor skill assessment tools may include the Alberta Infant Motor scale [30] or gross motor function measure [31]. These are general assessment tools and have not been validated for MBS but would provide baseline measures and allow progress to be mapped. Those patients with gross fine motor issues would be referred to community therapy teams for advice and input.

## 14. Sleeping

In our experience, sleep disturbance is common and characterised by poor-quality night-time sleeping with frequent arousal in addition to parasomnias [32]. There is little published evidence base for this association [33,34,35] and although little is known regarding the aetiology, the reticular formation involved in sleep–wake control is situated in the area of the brainstem affected by MBS.

Typically, younger children go to sleep normally, but wake after a few hours, remaining awake for the remainder night, with little sign of tiredness the next day. Parents should maintain sleep diaries documenting the sleep patterns their child adheres to and additionally ensure that the sleep environment is appropriate (comfort/temperature/light exposure, etc.). It is important to determine whether the MBS child is sleeping adequately to function during the daytime. If the amount of sleep meets their biological needs, then a pragmatic approach may need to be adopted where their sleep schedule is adjusted to meet the requirements of the child rather than adhering to set schedule to enforce a circadian rhythm. We suggest, when possible, the child is provided with an area that is safe for them to play in, with activities to keep them occupied while the rest of the family sleeps. The experience garnered from our centre suggests that sleep improves as MBS children grow.

Central sleep apnoea has been reported in several cases and polysomnography may be appropriate should this or other airway issues be suspected but is otherwise of limited utility. Obstructive causes mandate ENT review to exclude correctable causes. If the child is at risk of aspiration at night, it may be appropriate to improve sleep position by tilting the bed head up. Children who show any signs of nocturnal hypoventilation (difficulties wakening, morning drowsiness, poor appetite, hyperactivity, deteriorating behaviour, declining school performance, unexplained weight loss, morning headaches) should have either a formal sleep study or a TOSCA study (transcutaneous pCO_2_, SaO_2_, pulse).

## 15. Psychological Care

Two main groups of patients exist [4]—those who have typical cognitive development and present with concerns relating to visible difference and a group that have developmental issues and special educational needs. In both groups, screening questionnaires can be used to identify potential difficulties and concerns. Currently, the ASQ (ages and stages questionnaire; up to 4 years of age) and SDQ (strengths and difficulties questionnaire; parent-reported from age 4 and self-reported from age 11) are employed by us; they are well validated and widely used, but these are general tools and lack specificity to this patient group. There are no reliable outcome measures to date that cover all aspects of this condition. Assessments should be routinely offered in early life and then during periods of significant transition (entering school, transition to high school and leaving school) in order to identify issues relating to behaviour, attention and hyperactivity, bullying, peer relationships and emotional wellbeing, as well as at other points in the child’s life if specific concerns are raised.

Common areas of concern may include low self-esteem, bullying and low mood/increased anxiety related to visible difference, as well as potential difficulties expressing and communicating emotion via facial expression. It is imperative to assess a child’s peer relationships and any experiences of bullying, which, if present, could have a high impact on a person’s self-worth and associated mood difficulties throughout childhood and into adulthood. A measure for stigma among people with facial differences can be administered with older children with moebius [36], whilst younger children can be directly asked via anecdotal clinical interview. Other difficulties may be raised, including autism spectrum traits and difficulties associated with cognitive concerns/developmental delay. Whilst autism spectrum traits are recognised as a likely association, conclusive estimates of the prevalence of these traits within MBS have not been consistently identified in the research to date [37,38,39,40]. Generally, psychosocial interventions in MBS may focus on parental adjustment, psychoeducation, managing challenging experiences such as bullying, and specific therapies focused on low mood and anxiety management, including increasing self-compassion. Role-play and social skills training has been advocated [41] but is not in widespread use. This may be useful with children and families where social-communication needs have been identified. Specific therapeutic modalities such as cognitive-behavioural therapy and compassion-focused therapy may be used (particularly with parents and older children); however, there are likely to be limitations with the application of some of these therapies with children presenting with significant cognitive difficulties. Narrative therapy (such as writing a book on “My Mobius Syndrome”) has little evidence base, but anecdotally helps with understanding in the younger patient and can be useful tools to help build confidence. Psychologists and/or their colleagues should provide psychoeducation within the meobius patient’s school or other systems, to support teachers’ and others’ understanding of the condition. This would consequently support empathic management of the child including supporting peer relationships and social development. This could have considerable benefit on the child or young person’s social-emotional experience and development of self-esteem.

Transitions are important to consider and the Psychologist should routinely consider discussions around transitions into adulthood in lifespan services such as dating and relationships, further education and employment. This should include information from appropriate colleagues regarding the potential for employment discrimination due to facial difference of disability and to have access to associated legal protections, for example the UK Equality Act.

## 16. Genetics

As no common pathogenic variants have been identified for MBS, genetic testing is currently primarily directed at excluding alternative diagnoses. All patients with multiple structural abnormalities being investigated for possible MBS should undergo chromosomal microarray as the first-line genetic test. This will exclude alternative genetic conditions caused by currently detectable variants and can typically be requested by the physician leading the patient’s care.

A clinical geneticist should evaluate patients to undertake a detailed family history and dysmorphology assessment, primarily to exclude alternative genetic conditions (Table 2). Further testing to elucidate the underlying genetic cause is currently only available on a research basis and will use techniques such as whole-exome or whole-genome sequencing to look for putatively causative variants in genes such as *TUBB3*, *KIF21A*, *TUBB6*, *MYMK* and *PLEXIN-D1*/*REVL3*. Consideration should be given to entry into a clinical trial, e.g., the Moebius Syndrome Research Consortium in USA. Whole-genome sequencing is likely to become standard practice for the investigation of the child with Moebius syndrome who has a normal chromosomal microarray in the next one to two years.

## 17. Smile Surgery and Facial Therapy

Arguably, the most visible difference is the mask-like facies that hampers non-verbal communication: the child is unable to smile to indicate happiness and conversely cannot demonstrate sadness. Assessment of the facial nerve will usually demonstrate small areas of movement in the face but insufficient for facial expression. Electrophysiological testing is not necessary. Objective assessment is performed with photography; facial grading scales generally rely on a contralateral “normal” side, which is frequently absent [42,43]. If there is some movement, there is scope for physical therapy, which involves an individualised neuromuscular retraining approach, which may include stretches, massage and movement re-education (see Supplementary Materials) [44]. Mirror feedback can help those with some movement control their symmetry. However, physiotherapy alone will not be able to create movement where there is none. For this reason, surgical reconstruction of the smile has been long advocated [45]. Smile surgery involves the transplantation of a muscle (typically from the thigh) to the face using facelift incision and connecting the muscle to a branch of the trigeminal nerve for biting: This is termed a free gracilis muscle transfer powered by the masseteric nerve, which provides a predictable voluntary smile as this nerve is unaffected by the syndrome. Most recently, another approach is to power the muscle with any facial nerve branches that are active, which is a new concept in reanimating selected patients with this condition [46]. Facial rehabilitation following surgery allows the child to learn to use the muscle to smile and animate the face. Typically, surgery is offered after the age of 4 and the dogma is “the earlier the better” but patient-reported evidence for the psychological benefit is lacking. For this reason, a comprehensive psychological assessment with the child and parents is suggested and the senior author’s approach is to wait until the child can comprehend the issues, understand what the surgery can achieve and participate in pre- and post-operative physiotherapy.

## 18. Conclusions

1. Moebius syndrome is essentially a diagnosis of exclusion once other conditions have been ruled out. This requires specialist assessment and genetic workup.
2. Initial assessment is based on an ABC-style approach, with airway, feeding, vision and speech taking priority in the early years.
3. Speech and language support for oromotor skills in the early years and psychological support in later years are the mainstay of management.
4. The main time points for assessment after initial work up are during important life events: starting nursery, starting school, transition to high school, leaving high school.
5. Surgical interventions are geared towards specific functional problems.

## Figures and Tables

**Table 1:** Features associated with MBS (adapted from Bell, 2018) [4].

Feature	Percentage of Recorded Cases Positive for Feature (%)
**General features**	
Dysarthria	89.2
Abnormal motor coordination	85.5
Failure to thrive	79.1
Micrognathia	75.5
Hypotonia	75.4
Epicanthic folds	73.2
Developmental delay	70.3
Swallowing difficulties older	67.2
Brain imaging abnormality	65.6
Ears dysplastic or low set	65.4
Hypoplastic tongue	63
High palatal arch	61.7
Blepharoptosis	40.8
Cleft palate	37.7
Cardiac abnormalities	33.3
Conductive deafness	24.6
**Cranial nerve palsies**	
CN I	-
CN II	-
CN III	48.4
CN IV	37.5
CN V	25.6
CN VIII	30.8
CN IX	43.7
CN X	53
CN XI	21.7
CN XII	45.3
**Limb and skeletal anomalies**	
Any lower limb anomaly	62.4
Any upper limb anomaly	55.4
Talipes	53
Brachydactyly	47.4
Poland anomaly	38.2
Syndactyly	36
Scoliosis	34.3
Symbrachydactyly	20.5
Camptodactyly	16.9

**Table 2:** Differential diagnosis of Moebius syndrome.

**Genetic Diagnoses**
Hereditary Congenital Facial Palsy (*HOXB1*)
Carey–Fineman–Ziter syndrome (*MYMK*)
Genetic Moebius syndrome *PLXND1*/*REV3L*
**Neuromuscular Diagnoses**
Myasthenia gravis
Fascioscapulohumeral dystrophy
Congenital myotonic dystrophy
**Congenital cranial dysinnervation disorders**
Congenital fibrosis of the extraocular muscles (*KIF21A*/*TUBB3*/*TUBB6*)
Duane retraction syndrome

**Table 3:** Step-wise management of reflux and drooling.

**Reflux**
Alginates
Food thickeners
Proton pump inhibitors, e.g., omeprazole
Pro-kinetics, e.g., domperidone
**Drooling**
Posture management
Oral awareness and oral skills training
Orthodontic management and appliances
Pharmacotherapy
Botulinum toxin injections to salivary glands
Surgery to salivary glands

## Supplementary Materials

The following supporting information can be downloaded at: https://www.mdpi.com/article/10.3390/jcm13113309/s1, File S1: Auditory assessment and facial therapy management. References [47,48,49] are cited in the Supplementary Materials.
