# Supplementary material for: Multidisciplinary Care for Moebius Syndrome and Related Disorders: Building a Management Protocol
Source: J Clin Med. 2024 Jun 4;13(11):3309. doi: 10.3390/jcm13113309 (PMC11172851; doi:10.3390/jcm13113309)
Supplement: Supplementary file 1 [file jcm-13-03309-s001.zip › jcm-2976342-supplementary.pdf]

Journal of
Clinical Medicine

MDPI

Supplementary Materials: Auditory assessment and facial therapy management.

Auditory assessment

Hearing is critical for development of speech and the audiovestibular system may be affected in MBS [12,13] with conductive deafness or sensorineural dysfunction in up to 30% [4]. Most audiovestibular investigations are best performed when the child is older (approximately 8 years) and are achieved through objective and behavioral tests. All children in the UK have a neonatal (objective) hearing test (otoacoustic emissions) and if abnormalities are detected additional testing (eg. auditory brainstem response) is indicated. Behavioral testing relies on the child to communicate *their perception of hearing* and multiple factors in MBS may confound this. For this reason, a low threshold for objective testing and monitoring of expressive and receptive language skills will allow for interventions before hearing loss can contribute to speech delay [16].

Once hearing thresholds have been established any hearing loss can be appropriately rehabilitated according to the established hearing level; levels between 40-100dB require digital amplification to improve hearing. Profound hearing loss (>100dB) will require cochlear implantation. If there is cochlear nerve dysfunction such as aplasia, auditory brainstem implant can be performed with one example of this in MBS. [47].

Assessment of the vestibular system has not been studied in MBS, but may contribute to the poor motor coordination. In addition to history (supplementary material) and vestibulogram, a plethora of clinical vestibular tests can be performed; these have largely been replaced with quantifiable laboratory diagnostics.[48] Over the age of 8, CT can be used to assess for a dysmorphic bony labyrinth in all hearing loss/vestibular cases. Management through vestibular rehabilitation therapy can be customised accordingly and has proven benefit, though central vestibular processing difficulties are usually more complex and reliant on an MDT approach with neurosensory and neurophysiological therapies and cognitive neurotherapy.[49] Outcome measures in children are poor (eg. dizziness handicap inventory) so we ask parents to employ a balance diary and use this to guide management.

Taking an Auditory Assessment

History

- How is your child's speech?
- Does your child respond to verbal commands and day to day sounds?
- How is their behaviour? (withdrawn/overly active)
- Any difficulty hearing (high TV volume or shout to hear)
- Recurrent ear infections? (glue ear)
- Intolerance to loud sound (hyperacusis)

Investigations

- Pure tone audiometry
- Tympanometry
- Acoustic Reflex Threshold
- Oto-acoustic emissions
- Auditory brainstem response

Taking a Vestibular Assessment

History

- Based on surrogate (parental) history if <8 years
- But can report >8 years
- Ask the parent about motor development

- Difficult ambulation in the dark (do they fall?)
- Falling/tripping/clumsy?
- Brief lasting sudden drop attacks (<5 seconds)
- Difficulties in challenging visual environment
- Difficulties riding bike
- No loss of consciousness
- Inability to do quick head movements
- Difficulties in climbing stairs and lifts
- Travel sickness and fear of heights

Vestibular physical exam

- Pursuit and saccadic system
- Mastoid vibration test
- Head impulse test
- Head heave test
- Head shake test
- Dynamic visual acuity test
- Ocular counting roller test
- Lateral flexion test
- Detection of nystagmus at rest
- Rombergs test
- Unterburger test
- Tandem gait

Vestibular laboratory exam

- Video-nystamo-graphy (VNG) with and without optic fixation
- Video head impulse test
- Vestibular evoked myogenic potential test (VEMP)
- Rotatory chair test

Facial Therapy

Assessment

Photographs and physical examination

- No innervation/no movement = no role for facial therapy
- If evidence of some movement/innervation = rehab

Treatment – if no movement – no rehab needed

- Retraining with or without biofeedback or visual mirror feedback
- Stretching – working on symmetry for any contralateral dominance (dampen this to improve aesthetic symmetry)

NMR (neuromuscular retraining)/Mirror therapy

- Assess goals: what does patient hope to achieve?
- Treatment geared towards movement present + what may be achieved

Initial therapy – movement exercises at home

- Review some weeks after
- SALT input

Surgery Facial Therapy

Preop – assessment as above

- Biting teaching practice (gracilis to masseter)

- Understanding of therapy needed

Post op

- Seen on ward to mobilise, assess swelling help scar management.
- Exercise for movement – massages/stretches to hold the strength
- Scar management
- Re-education assist with retraining smile
- Stretching for contralateral dominance
- Scar massage
- Moebius – signposting for community physiotherapists
- App based games to encourage movement
